# Asymptomatic diaphragmatic rupture with retroperitoneal opening as a result of blunt trauma

**DOI:** 10.4103/0974-2700.66556

**Published:** 2010

**Authors:** Adnan Narci, Tolga Altuğ Şen, Reşit Köken

**Affiliations:** Department of Pediatric Surgery, Faculty of Medicine, Afyon Kocatepe University, Afyonkarahisar, Turkey; 1 Department of Pediatrics, Faculty of Medicine, Afyon Kocatepe University, Afyonkarahisar, Turkey

**Keywords:** Blunt trauma, child, diaphragmatic rupture

## Abstract

Blunt traumas of the abdomen and thorax are important clinical problems in pediatric ages. Severity of trauma may not always be compatible with the patients’ clinical situation. A 2-year-old male child was admitted to our emergency clinic as a result of tractor crash accident. Physical examination of the child was normal. The abdominal and thoracic ultrasonography (USG) examination performed in the emergency clinic was normal. In thoracic computed tomography (CT) scan of the patient, there was irregularity of the right diaphragmatic contour that was described as micro perforation-rupture (the free air was just in the perihepatic and retroperitoneal area, which was not passing through the abdomen). The patient was followed-up for 1 week in the hospital with a diagnosis of retroperitoneal diaphragmatic rupture. It is not appropriate to decide the severity of trauma in childhood on the basis of clinical findings. Although severe trauma and sustaining radiological examinations, the patients’ clinical pictures may be surprisingly normal, as in our patient. In such cases, there may not be any clinical symptom. CT scan examination must be preferred to USG for both primary diagnosis and follow-up of these patients. According to the current literature, there is no reported case with retroperitoneal rupture of the diaphragm.

## INTRODUCTION

Diaphragmatic injuries are rarely seen in children and accurate diagnosis is difficult because of the paucity of clinical symptoms and thus those injuries may be easily overlooked.[1,3] In contradiction to adulthood traumas, in children, the clinical picture does not reflect the severity of trauma. For the accurate and duly diagnosis of those patients, the physicians must suspect the severity from the diaphragmatic rupture (DR), as any delay in the diagnosis may lead to significant morbidity and mortality. The mechanism of injury in these accidents may be simply explained as follows: because the body quickly decelerates whereas, at the same time, the thoracic and abdominal organs continue to move downward and forward with maximal velocity, a tear of the vessels and tissues from the points of attachments is caused. In this case report, we aimed to present a case with a DR who had radiological and clinical features that were not described before.

## CASE REPORT

**Figure 1:**
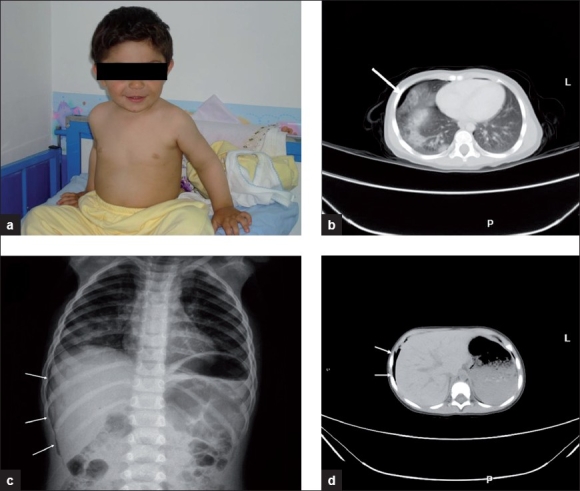
(a) The patient seems to be good shortly after the accident. (b) Severe parenchymal contusion at the right lung and minimal contusion at the left lung and bilateral minimal pneumothorax are seen. (c and d) Free air is just in the perihepatic and retroperitoneal area and not passing into the abdomen

A 2-year-old male child was admitted to our emergency clinic as a result of tractor crash accident. The patient fell from a moving tractor and the back wheel of the tractor had passed over the child’s thorax. There was no pathologic finding in the physical examination. Although the patient appeared well and we did not suspect a serious injury, we performed all standard emergency diagnostic procedures. The abdominal and thoracic ultrasonography (USG) examination in the emergency service was normal. Thoracic computed tomography (CT) scan revealed severe parenchymal contusion at the right lung and minimal contusion at the left lung, bilateral minimal pneumothorax, irregularity of the right diaphragmatic contour that was described as micro perforation-rupture (the free air was just in the perihepatic and retroperitoneal area and not passing through the abdomen) and a hypodense area compatible with contusion on the liver and spleen [Figure 1]. The patient was followed-up for 1 week in the hospital with the diagnosis of retroperitoneal DR. The patient’s vital signs were stable and laboratory tests were normal during treatment. The patient did not require any surgical intervention. The control CT scan was normal. The patient was discharged from the hospital without any complications.

## DISCUSSION

Although reported cases of DR in children are rare in the literature, and because motor vehicle accidents are increasing day after day, the number of reported cases tends to increase. There are several mechanisms leading to diaphragm rupture after a blunt trauma. These mechanisms are avulsion of the attachments of the diaphragm or shearing of the stretched membrane after right or left lateral impact to the chest wall, rib fracture fragments directly penetrating the diaphragm and a sudden increase in the intraabdominal pressure throughout the abdomen with the relatively weak, unprotected left diaphragm tearing from the force. The right hemidiaphragm is also protected from abdominal impact by the energy-absorbing liver.[4,5]

Diagnosis of DR is difficult in children due to the paucity of clinical symptoms. In children, the severity of trauma may not be determined as easily as in adults. Hemidiaphragmatical rupture more commonly involves the left side and occurs in 56–86% of the ruptured cases. Right-sided tears occur in 11–39% of the cases and bilateral and other sites account for 2.4–13.0% of the cases.[4,6] In our patient, the DR was on the right side. Physical examination is rarely useful in the multitrauma scenario. The chest radiography for initial screening of DR has a relatively low sensitivity for diagnosis, which was reported as diagnostic in 46% of the patients with rupture of the left side and suspicious for DR in another 18%.[7] Most of the authors suggested that the preferred diagnostic method should be CT in blunt traumas. Conventional CT has a variable sensitivity of 14–61% and a specificity of 76–99% in the diagnosis of DR.[1] Helical CT is a more accurate diagnostic method in the detection of DR, with a sensitivity of 71–84% and a specificity of 77–100%.[5] However, the sensitivity for diagnosis of right-sided rupture is not as high as for the left side, with a CT sensitivity and specificity of left hemidiaphragm rupture reported as 78–100% and 100%, respectively, and the sensitivity and specificity of right hemidiaphragm rupture reported as 50–83% and 100%, respectively.[4] Although CT is more sensitive and specific than the other diagnostic techniques (e.g., X-ray, USG), diaphragmatic injuries, especially those on the right side, most frequently have remained undiagnosed on CT during the acute evaluation of trauma patients.[7,8] In our patient, we preferred USG examination initially. The USG examination of the patient was normal. In the CT scan, there ware findings of major trauma that were explained before.

Associated injuries are common in traumatic rupture of the diaphragm due to the relatively large force required to disrupt the diaphragm, concurrently damaging the adjacent organs. Associated major injuries are reported in 52–100% of the patients. The most commonly damaged intraabdominal organs are the liver, reported as being disrupted in 93% of the patients with right-sided injury, and spleen in 24% of those with left-sided injury. In all cases of diaphragm tears, splenic injuries occur in 27–63% of the cases. Other commonly associated abdominal injuries include pelvic and renal injuries and associated intrathoracic injuries include hemopneumothorax and rib fractures.[6–10] There were some associate injuries in our patient (e.g., liver and spleen contusion, bilateral pneumothorax), but hemothorax or rib fracture was not present.

Open surgical repair has been the traditional method of treating blunt traumatic diaphragmatic injuries. Surgical approach is most often abdominal in acute cases, but may require a thoracic approach, especially with right-sided injury. Mesh or prosthetic repair is rarely needed in the acute stage, but may be useful for a delayed repair. Laparoscopy may be a useful technique when standard diagnostic methods fail to reveal a DR and it is also useful for management. We did not apply any surgical procedure in our patient because the rupture was minimal and the patient was not deteriorated. We closely followed him up for 1 week and performed the control CT, which was normal.

## CONCLUSION

It is not appropriate to decide the severity of trauma in childhood on the basis of clinical manifestations alone. In addition, we asserted that CT examination of patients must be preferred to USG examination for both primary diagnosis and follow-up of these patients.
